# Supplementary material for: Resolving the full spectrum of human genome variation using Linked-Reads
Source: Genome Res. 2019 Apr;29(4):635–45. doi: 10.1101/gr.234443.118 (PMC6442396; doi:10.1101/gr.234443.118)
Supplement: Supplemental Material [file supp_gr.234443.118_Supplemental_longranger-2.2.zip › longranger-2.2.tar.bz2!/lr2.2-public/lib/stan/cmdstan/stan/doc/api/html/print__stack_8hpp_source.html]

Stan: src/stan/agrad/rev/print\_stack.hpp Source File


|  |  |
| --- | --- |
|  | Stan  2.5.0  probability, sampling & optimization |


- Main Page
- Related Pages
- Namespaces
- Classes
- Files

- File List
- File Members

All Classes Namespaces Files Functions Variables Typedefs Enumerations Enumerator Friends Macros Pages

- src
- stan
- agrad
- rev

print\_stack.hpp

Go to the documentation of this file.

1 #ifndef STAN\_\_AGRAD\_\_PRINT\_STACK\_HPP

2 #define STAN\_\_AGRAD\_\_PRINT\_STACK\_HPP

3

4 #include <ostream>

5 #include <stan/agrad/rev/var\_stack.hpp>

6 #include <stan/agrad/rev/vari.hpp>

7

8 namespace stan {

9  namespace agrad {

10

20  inline void print\_stack(std::ostream& o) {

21  o << "STACK, size=" << var\_stack\_.size() << std::endl;

22  for (size\_t i = 0; i < var\_stack\_.size(); ++i)

23  o << i

24  << " " << var\_stack\_[i]

25  << " " << (static\_cast<vari\*>(var\_stack\_[i]))->val\_

26  << " : " << (static\_cast<vari\*>(var\_stack\_[i]))->adj\_

27  << std::endl;

28  }

29

30  }

31 }

32 #endif

var\_stack.hpp

vari.hpp

stan::agrad::var\_stack\_

std::vector< chainable \* > var\_stack\_

**Definition:** var\_stack.cpp:13

stan::agrad::print\_stack

void print\_stack(std::ostream &o)

Prints the auto-dif variable stack.

**Definition:** print\_stack.hpp:20


---

[ Stan Home Page ]

*© 2011–2014,
Stan Development Team.*
